# RISK AND RESILIENCE RELATED TO SELF-PERCEPTIONS OF AGING AMONG LESBIAN AND GAY OLDER ADULTS

**DOI:** 10.1093/geroni/igae098.1206

**Published:** 2024-12-31

**Authors:** M Aaron Guest, Hannah Giasson, Rachel Koffer

**Affiliations:** Arizona State University, Phoenix, Arizona, United States; Arizona State University, Tempe, Arizona, United States; Arizona State University, Phoenix, Arizona, United States

## Abstract

Lesbian and gay individuals may face unique experiences and expectations associated with growing older. In part, this may result from the perception of an overemphasis on youth in the LGBTQ+ community. The present study aimed to provide a preliminary model of how lesbian and gay adults view their aging and identify psychosocial risk and resilience factors specific to this community. Data are from a pilot study of social perceptions of aging in lesbian and gay adults, in which 45 gay and 46 lesbian adult participants (ages = 55-87, 70% white) completed questionnaires on their health, perceptions of aging, community engagement, identity congruence, and stressful experiences. Self-perceptions of aging were measured with an 8-item scale ranging from 1 to 6, where higher scores indicate greater agreement with positive perceptions of aging. In a regression model accounting for age, self-rated health, subjective socioeconomic status, and race/ethnicity, lesbian women reported better self-perceptions of aging than gay men (b = 0.50, SE = 0.16, p <.01). Further, greater outness was associated with better self-perceptions of aging (b = 0.04, SE = 0.01, p =.01), LGBTQ+ community engagement did not relate to self-perceptions of aging (b = -0.11, SE = 0.12, p =.38). More stressful experiences in daily life were associated with worse self-perceptions of aging (b = -0.14, SE = 0.05, p <.01). Results will be discussed in the context of a model of risk and resilience for “accelerated social aging” among Lesbian and Gay populations.

